# Psychosocial and socioeconomic burden of vasomotor symptoms in menopause: A comprehensive review

**DOI:** 10.1186/1477-7525-3-47

**Published:** 2005-08-05

**Authors:** Wulf H Utian

**Affiliations:** 1 North American Menopause Society, 5900 Lander Brook Drive, Mayfield Heights, OH 44124, USA

## Abstract

Many women experience vasomotor symptoms at or around the time of menopause. Hot flushes and night sweats are considered primary menopausal symptoms that may also be associated with sleep and mood disturbances, as well as decreased cognitive function. All of these symptoms may lead to social impairment and work-related difficulties that significantly decrease overall quality of life. Hot flushes have shown a great deal of variability in their frequency and severity in women. In some women, hot flushes persist for several months; in others, they may last for more than 10 years. Traditionally vasomotor symptoms were reported to begin 5 to 10 years prior to the cessation of the final menstrual cycle, corresponding with the initial decline in circulating gonadal hormones; however, night sweats in particular most often begin in perimenopause. The pathogenesis of hot flushes has not yet been fully elucidated, but the circuitry involving estrogen and neurotransmitters, norepinephrine and serotonin specifically, are hypothesized to play a major role in the altered homeostatic thermoregulatory mechanisms underlying these events.

Menopause-associated vasomotor symptoms are associated with significant direct and indirect costs. Overall costs of traditional pharmacotherapy or complementary and alternative medicine modalities, including over-the-counter treatments and dietary supplements, for managing menopause-related vasomotor symptoms are substantial and include initial and follow-up physician visits and telephone calls. Additional costs include laboratory testing, management of adverse events, loss of productivity at work, and personal and miscellaneous costs. Pharmacoeconomic analyses, including those that consider risks identified by the Women's Health Initiative, generally support the cost-effectiveness of hormonal therapy for menopause-associated vasomotor symptoms, which have been the mainstay for the management of these symptoms for more than 50 years. However, because many women now want to avoid hormone therapy, there is a need for additional targeted therapies, validated by results from controlled clinical trials that are safe, efficacious, cost-effective, and well tolerated by symptomatic menopausal women.

## Review

Menopause is characterized by physiologic and psychosocial changes in a woman's life. Menopause may be associated with vasomotor symptoms (VMS; hot flushes [also referred to as hot flashes] and night sweats), bone loss, urogenital atrophy, urinary tract infections and incontinence, increased cardiovascular risk, somatic symptoms, sexual dysfunction and decreased libido, and loss of skin elasticity. VMS, and the sleep and mood disturbances that often result from them, can have a significant negative impact on overall quality of life (QOL) for a substantial number of women. The impact of VMS has gained in importance as the lifespan of women has increased throughout the world since women can expect to spend a significant portion of their lives after menopause. This period should be a highly productive time for women, and maintaining functional ability and a good QOL is of utmost importance. Accordingly, it is important to understand the economic and QOL impacts of menopausal VMS as well as the most recent pharmacoeconomic analyses of different approaches to managing symptoms. Thus, the aims of this paper are to briefly review the epidemiology of VMS, describe what is known about the physiologic basis of these symptoms, and examine the global and health-related quality of life (HRQOL) effects of VMS in women, with a focus on psychosocial and economic impairments, and costs associated with treatments.

## Epidemiology of VMS

### Prevalence and risk factors

Recent US Census Bureau statistics indicate that approximately one third of women are older than 50 years of age [1]. It is estimated that 75% of women in this age group will experience hot flushes, a value supported by a recent longitudinal study of 454 women who were followed from premenopause to postmenopause [2]. Thus, in the United States alone, there are approximately 40 to 50 million women who experience hot flushes [1]. Worldwide, between 50% and 85% of women (approximately 360 million) older than 45 years of age experience hot flushes [3]. The prevalence of hot flushes varies widely across populations and is strongly influenced by culture and ethnicity. In the United States, the Study of Women's Health Across the Nation (SWAN) surveyed more than 16,000 women and found that the prevalence of hot flushes was highest among African Americans (46%), followed by Hispanics (34%), whites (31%), Chinese (21%), and Japanese (18%) [4]. In other parts of the world, rates of hot flushes vary widely as well, with the lowest prevalence observed in China (10%) and other Asian nations [5].

Many attempts have been made to identify demographic characteristics associated with a significantly increased risk of hot flushes. For many years, low body mass index (BMI) and race were considered significant predictors of VMS, with thin, white women believed to at the highest risk for hot flushes. More recent findings have suggested that high BMI and African American race are associated with a higher risk of VMS. This shift may be related to better sampling of the general population by major clinical trials because, traditionally, white middle-class women participated in clinical trials that often did not include women from other ethnic groups. The multiethnic SWAN not only demonstrated a link between an elevated BMI (≥27 kg/m^2^) and hot flushes [4], but an increased prevalence in African American women, as mentioned. Ongoing studies continue to investigate potential predictors of hot flushes. Smoking, maternal history, history of premenstrual complaints, elevated basal core body temperature, low physical activity, low socioeconomic status, and low levels of estrogen and high levels of luteinizing and follicle-stimulating hormones prior to the menopausal transition have all been associated with an increased risk of hot flushes [4,6-8].

### Timing of hot flushes

The timing and frequency of hot flushes have been reviewed by several researchers [9]. SWAN demonstrated that hot flushes occur earlier than previously believed and may become less frequent and less intense as menopause progresses. SWAN data indicated that VMS were more frequently reported by women in late perimenopause with a relative risk for hot flushes at 1.0 during premenopause (the 1 or 2 years prior to menopause), 2.06 during early perimenopause (the early menopausal transition), 4.32 during late perimenopause (the late menopausal transition), and 2.81 during postmenopause [4]. The frequency of hot flushes varies but tends to remain consistent for an individual [10]. Many women have hot flushes on a daily basis, some as frequently as every hour, whereas others have VMS infrequently (ie, weekly or monthly) [10]. The majority of women experience hot flushes for 6 months to 2 years, with the highest number of women reporting symptoms during the first 2 postmenopausal years [9,10]. However, in another study, 26% of women reported having hot flushes for 6 to 10 years, whereas 10% have had VMS for more than 10 years [11].

### Pathophysiology

The cause of hot flushes has yet to be determined because of the limited research focus in this therapeutic area. Hot flushes are believed to result from the brain's response to diminished hormones and hormonal fluctuations that occur during the menopausal transition. Ovarian hormones have been shown to influence thermoregulatory mechanisms that regulate temperature homeostasis in the hypothalamus. The neurotransmitters serotonin and norepinephrine play a role in modulating core body temperature, neurochemical messaging, and peripheral vasculature [12]. Kronenberg and colleagues were the first investigators to document cardiovascular, temperature, hormonal, and autonomic parameters with hot flushes and link them with thermoregulatory mechanisms [13], with more recent mechanistic information published by Freedman [14] and Deecher et al. [12].

### Effects of associated VMS on QOL

Perceived QOL is difficult to measure and there is no universal agreement on how it should be quantified. Objective measurements of health status (often referred to as HRQOL) may not capture the patient's perception of overall life satisfaction. QOL can be defined as a reflection of an individual's belief about functioning and achievement. HRQOL may be viewed as the individual's perception regarding her physical, cognitive, and mental health as well as social situation [15]. Assessments of overall QOL for menopausal women must include consideration of somatic symptoms (hot flushes, night sweats, urogenital atrophy), psychological symptoms (depression, mood swings, irritability, anxiety), and life circumstances (function in the workplace). Thus, overall QOL may include four major factors: occupational, health-related, sexual, and emotional [15]. Consideration of HRQOL is also influenced by women's increased risk of multiple chronic diseases associated with menopause, including osteopenia, osteoporosis and related fractures, and cardiovascular disease [16].

### VMS-related effects on QOL

VMS can have a significant negative impact on QOL in younger and older women, contributing to physical as well as psychosocial impairment (Table 1). Becoming flushed and sweating profusely in a social or work-related situation may cause extreme anxiety for many women and lead to social isolation [17].

**Table 1:** Vasomotor Symptoms and Related Psychosocial Impairment During the Menopausal Transition

**Hot flushes**
**Night sweats**
**Sleep disturbances**
Insomnia
Sleep apnea
**Mood swings**
Irritability
Sadness
Tension
**Cognitive deficits**
Poor concentration
Verbal memory problems
**Social impairment**
Disruption of family relationships
Social isolation
**Work-related difficulties**
Reduced productivity
**Other Quality-of-life impairment**
Embarrassment
Anxiety
Fatigue

Although it is generally accepted that VMS are troubling to many women and adversely impact their QOL, these effects are difficult to quantify because of the many factors that contribute to overall QOL satisfaction. For example, an objective of the Women's Health Initiative (WHI) was to determine whether hormonal therapy (HT) could reverse impaired HRQOL in 16,608 women aged 50 to 79 years. Results from this analysis indicated that estrogen plus progesterone did not yield any significant benefits in any of the HRQOL outcomes when compared with placebo over 3 years of follow-up [18]. However, this investigation did not examine global QOL in relation to VMS as an a priori outcome, and the women studied were not only excluded if the investigators felt they were having significant VMS that would interfere with long-term study involvement, but were older than those who typically have VMS and use HT. Another limitation was the use of a QOL questionnaire that may have been inadequate for determining the global impact of VMS. More recently, a new QOL scale has been designed specifically for the perimenopausal population [19]: The Utian Quality of Life (UQOL) Scale evaluates occupational, health, emotional, and sexual QOL. This 23-item assessment should increase the reliability of QOL measurement in perimenopausal and postmenopausal women.

The physiologic changes associated with menopause often result in increased anxiety and stress. These feelings may arise from sleep deprivation, mood swings, and unpredictable hot flushes. Before menopause, most women have a monthly hormonal rhythm. When the cycle becomes disrupted by erratic hormonal fluctuations, a woman's sense of well-being can be disturbed. These changes also tend to occur at a time when women are more likely to experience other life changes, including divorce, widowhood, children leaving home, concerns about aging parents, and other caregiving issues. Self-image is another important variable, and women with poor self-images have more flush-related distress [20]. In combination, these factors may each contribute to a reduced global QOL as well as decreased work productivity and difficulties with personal and social relationships.

A large number of studies have documented the negative impact of menopause on QOL. Ledesert and colleagues studied a cohort of 289 women aged 45 to 52 years who were no longer menstruating and reported lower HRQOL on several measures of the Nottingham Health Profile compared with values for menstruating women [21]. A population-based study evaluated the effects of various medical conditions on work impairment in more than 16,500 individuals [22]. Menopause was one of the factors associated with significant work limitations. The impact of VMS on HRQOL has also been studied by Bobula and colleagues, who evaluated 1,655 healthy, nonhysterectomized, postmenopausal women ranging in age from 40 to 65 years who were not receiving HT [23]. Their results indicated a significant correlation between moderate to severe hot flushes and decreased QOL. Women with moderate to severe hot flushes had significantly poorer scores than women with no hot flushes on four of the eight 36-item Short Form Health Survey (SF-36) subscales (vitality, bodily pain, social function, and role limitations-emotional), the mental composite score, and the Women's Health Questionnaire (WHQ), with pronounced differences for VMS, sleep problems, sexual behavior, and somatic symptoms. Women with moderate to severe hot flushes also had significantly poorer scores than women with mild hot flushes on two of the eight SF-36 subscales (vitality and social function) and five of the nine WHQ domains, including VMS, sleep problems, sexual behavior, somatic symptoms, and depressed mood. Finally, women with mild hot flushes had significantly worse scores than women with no hot flushes on two of the eight SF-36 subscales (vitality and role limitations-emotional) and on the WHQ for VMS, sleep problems, somatic symptoms, memory/concentration, and menstrual symptoms.

### Impact of VMS on sleep, mood, and cognitive function

Despite the lack of agreement in the medical literature about the relationship between VMS on sleep quality, mood variability, and cognitive function, these symptoms are, in fact, primary complaints of menopausal women to their healthcare practitioners; as such, they are addressed in this review.

### Sleep disturbances

The causes of menopause-related sleep disturbances are controversial. It has been suggested that problems with sleep may occur in older women independently of menopause. For example, nocturia increases with age and may disturb sleep [24]. Depression, stress, and other factors (eg, restless leg and other periodic limb movement syndromes) may also contribute to sleep disturbances in these patients [25].

Sleep disturbances also have been specifically related to hormonal changes that trigger hot flushes or night sweats, independent of age. A National Sleep Foundation poll of 1,000 women between the ages of 30 and 60 years found that 36% of perimenopausal, postmenopausal, and oophorectomized women experienced hot flushes during the night [26]. In this study, 44% of women who experienced VMS while sleeping were perimenopausal versus 28% of women who were postmenopausal. The poll also showed that menopausal and postmenopausal women slept less than premenopausal women. According to the National Sleep Foundation, women with night sweats experienced an average of three occurrences per week. These events disrupted sleep and led to daytime irritability [26].

Menopause-related VMS also may be associated with insomnia and disordered breathing at night. More perimenopausal and postmenopausal women than menstruating women have difficulty falling asleep, staying asleep, and achieving refreshing sleep [26]. Insomnia symptoms in women in the various stages of menopause include difficulty falling asleep (29%) and early awakening with an inability to fall back to sleep (21%). Respiratory abnormalities also may contribute to sleep disturbances in menopausal women. Results from a study of 589 women indicated that those in the menopausal transition were at a greater risk for complaints of sleep apnea and hypopnea than were younger women [27]. Postmenopausal women had a 2.6-to 3.5-fold greater rate of sleep-disordered breathing than their premenopausal counterparts [27]. Because sleep complaints are part of menopause-associated VMS, disordered breathing is often overlooked as a potential cause of menopause-associated sleep disturbance [27]. Although these and other results have suggested a correlation between the occurrence of hot flushes and sleep complaints in menopausal women, only a few studies have employed objective methods for sleep evaluation (eg, polysomnography, actigraphy, quantitative electroencephalographic analysis). Results from these assessments have indicated that hot flushes correlate with the occurrence of objectively demonstrable sleep disruption in at least some women [28].

Inadequate and unrefreshing sleep can have many consequences. Over time, disruption of sleep secondary to hot flushes and/or night sweats leads to chronic sleep deficits, significantly impaired alertness and mental acuity, carelessness, forgetfulness, and decreased work productivity. In some cases, night sweats can drench bedclothes and sheets, further disrupting sleep and necessitating a change of clothes and covers, which can also disturb the sleep of the individual's bed partner. Thus, lack of sleep, tiredness, and irritability can affect daytime productivity as well as familial and social relationships.

### Mood

Menopause-associated changes in mood may result from a wide range of variables, including elevated sensitivity to environmental events secondary to decreased hormonal levels, changes in socioeconomic and/or marital status, culture, lifestyle factors, level of education, and history of depressive symptoms [29,30]. Longitudinal and cross-sectional studies carried out to date have not indicated a consistent relationship between the menopausal transition and increased risk of mood disorders [31,32]. However, results from the prospective population-based Melbourne Women's Midlife Health Project, which followed 438 women for 11 years and used the Center for Epidemiologic Studies Depression Scale to measure depression, indicated that depression scores were highest for women who were in the menopause transition stage (ie, had not reached their final menstrual period) or who had experienced surgical menopause. Current use of HT was associated with lower Center for Epidemiologic Studies Depression Scale scores (ie, less severe depressive symptoms) in this cohort [33]. These epidemiologic results are consistent with those from a small-scale clinical trial that demonstrated the significant benefit of short-term HT in perimenopausal women with depression [34]. It is important to note that many studies investigating mood and VMS have used depression scales. There are important distinctions between mood variability and major depression. It is incorrect to interpret the results from depression and simply extrapolate these findings to mood. Hopefully, new studies will address this issue and develop specific scales to delineate the impact of VMS on mood.

### Cognitive decline

Memory impairment is directly related to hot flushes in women who have undergone oophorectomy, but natural menopause itself does not necessarily result in significant cognitive dysfunction [35]. During a hot flush, blood flow decreases in the hippocampus, possibly impairing memory and cognition [35]. It has been suggested that such reductions in blood flow may contribute to the decreased mental clarity and short-term verbal memory problems experienced by many perimenopausal and postmenopausal women [35]. The importance of estrogen in cognition has been demonstrated by Jacobs and colleagues, who measured cognitive function and verbal memory in 727 older women (average age, 74.2 years). Study results demonstrated that cognitive test scores and verbal memory were superior in the women who received HT compared with those who did not [36].

### Costs associated with VMS and their treatment

**Figure 1:**
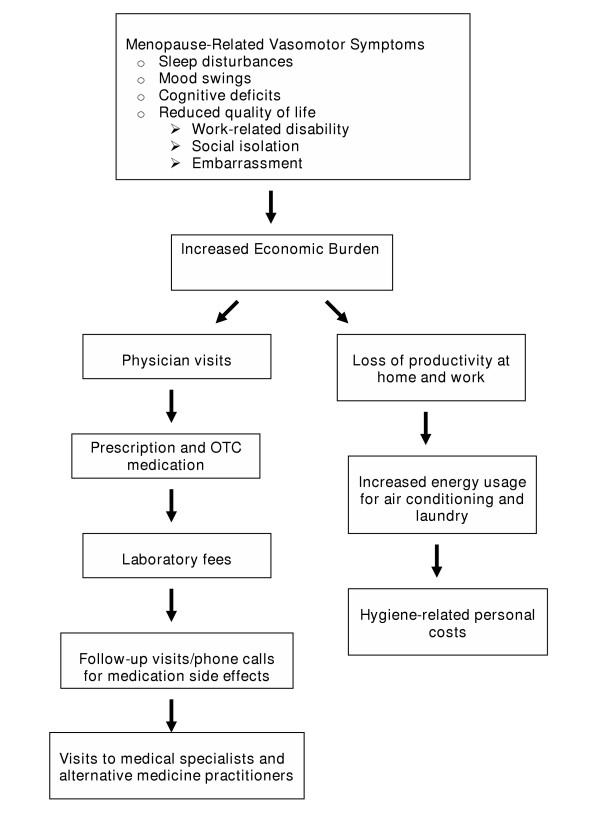
Flowchart of factors affecting management costs of menopause related vasomotor symptoms.

In the year 2000, there were approximately 50 million women aged 45 to 60 years in the United States; as life expectancy increases, that number will increase [1]. Many of these women will use the health care system for premenopausal, perimenopausal, and postmenopausal needs. These requirements pose a high economic burden on the women themselves and on the health care system (Figure 1).

Determining the cost-effectiveness of treatments aimed at relieving VMS is complex. Analysis must consider the direct costs of treatment, costs associated with treatment-related adverse events, and the health care costs saved with effective therapy [37,38]. Cost-utility analysis of treatment for VMS also considers the impact of treatment on improvements in QOL associated with the alleviation of symptoms. This impact of treatment is expressed in quality-adjusted life years (QALYs) [39].

Since the publication of results from the WHI in 2002, therapy for menopausal symptoms has undergone a dramatic transition. A marked decline has been seen in prescriptions for oral estrogen and HT. Prescriptions for oral estrogens declined from 56.8 million in 2001 to 37.2 million in 2003. The respective values for oral HT were 24.0 and 10.3 million [40]. However, the pendulum has swung from fear to a greater understanding of the risks and benefits of HT [41].

Costs incurred for the management of menopause-related VMS include visits to physicians; follow-up visits and telephone calls for the management of medication-related side effects and changes in medication; self-prescribed over-the-counter remedies, including complementary alternative medications (CAMs); HT; laboratory tests; lost productivity at work; personal costs for hygiene-related supplies; energy costs for the increased use of air conditioning; and extra laundry requirements for clothing and bed sheets soiled with sweat. A recent Gallup poll of menopausal women showed that hot flushes and night sweats were the first-and second-ranked symptoms that prompted physician visits. Among the women surveyed, 70% complained of hot flushes, 68% complained of night sweats, 50% had mood changes and moodiness, and 49% experienced insomnia and sleeplessness [42]. The costs associated with many of these symptoms have not been quantified, but they undoubtedly result in a significant burden for the women who experience them.

Before seeking medical advice at the onset of VMS, women are likely to obtain information from their peers, family members, or the Internet. In many cases women resort to self-diagnosis and treatment. They may combine over-the-counter drugs with medications prescribed for other conditions (eg, analgesics for headache; anxiolytics and antidepressants for anxiety, tension, and mood changes; sedatives/hypnotics for insomnia). Most of these treatments fail to provide significant relief of VMS, and many women ultimately consult their physicians after these remedies fail. Physician visits for VMS include an initial visit to a primary care physician and, potentially, follow-up visits to a gynecologist or CAM specialist. Subsequent visits and telephone calls are often needed for medication adjustment, laboratory testing, and managing side effects. In addition, women may seek counseling from a psychologist or psychiatrist for mood changes, insomnia, and difficulties with family and social interactions. Women may also visit a neurologist for relief from headaches and/or help with cognitive deficits. All of these factors add to the economic burden of VMS.

## Economic burden of therapy for symptom resolution

### Hormonal therapy

The economic burden of VMS management remains high even with the use of HT. Costs associated with HT include one or two visits for diagnosis and medication prescription as well as follow-up visits and telephone calls to manage side effects and evaluate the efficacy of therapy. Serious, but rare, adverse events associated with HT can lead to exceptionally high acute and chronic costs [43]. Evaluation and management of more common transient adverse events, including vaginal and uterine bleeding, breast discomfort, and breast nodularity, can also add significantly to the overall cost of HT for menopause-related symptoms. Approximately one third of patients who use HT switch to another form of therapy or make medication adjustments because of adverse events or compliance problems, increasing the overall cost of therapy [43].

Despite the complexities associated with determining the cost-effectiveness of HT for the treatment of women with menopause, several pharmacoeconomic analyses support the use of such treatment. Results from an early evaluation by Weinstein suggested that HT was cost-effective in menopausal women with prior hysterectomy or osteoporosis, but not in asymptomatic women with an intact uterus. The evaluation included information about the risks of endometrial cancer, uterine bleeding, and gallbladder disease as well as the benefits associated with the relief of menopausal symptoms and prevention of osteoporosis and fractures [44].

Although long-term continuous-combined HT (0.625 mg/day of conjugated estrogens plus 2.5 mg of medroxyprogesterone [CEE/MPA]) has been associated with the potential for increased health risks in some women, therapy limited to ≤5 years is no doubt beneficial for decreasing VMS, somatic symptoms, and resultant sleep and mood disturbances in women who experience bothersome menopausal symptoms. Moreover, short-term, low-dose therapy may minimize the risk of adverse effects associated with longer-term HT [45]. Botteman and colleagues compared the cost-effectiveness of short-term CEE/MPA 0.625/2.5 mg against that of norethindrone acetate 1 mg and ethinyl estradiol 5 μg (NA/EE), another continuous-combined HT with a different side-effect profile, and no interventional therapy for the management of moderate to severe VMS [45]. Anticipated 1-year baseline costs for the management of VMS included drug acquisition costs ($357 for NA/EE vs $474 for CEE/MPA), initiation of therapy requiring two physician visits ($132), HT-related breakthrough bleeding requiring endometrial biopsy and associated visits and laboratory testing ($345), or spotting requiring a telephone call to the physician ($16), and two physician visits for VMS plus clonidine therapy ($162; Table 2) [45]. Results from this analysis indicated that NA/EE was less expensive and more effective than CEE/MPA in QALYs. The cost-effectiveness of NA/EE was greater for patients with severe VMS than those with mild symptoms. NA/EE also was more cost-effective than no treatment, unless the symptoms were so mild that the discomfort of spotting or bleeding (the most common significant adverse effects of short-term HT) offset the QOL improvements associated with HT [45]. It also should be noted that the health risks associated with shorter-term (1 to 5 years) HT for the treatment of VMS are not known. Another trial evaluating NA/EE against CEE/MPA 0.625/2.5 mg or no therapy in premenopausal and perimenopausal women indicated that NA/EE is cost-effective as first-and second-line therapy [46]. Results from this study showed that NA/EE increased costs and QALYs compared with CEE/MPA and no therapy. Despite the increase in direct costs over no therapy and CEE/MPA, the cost effectiveness of NA/EE compared to no therapy is based on a decrease in the cost of treating menopausal symptoms, vaginal bleeding, and hip fractures and assumes a substantial increase in compliance compared to CEE/MPA as a result of improved control of bleeding with NA/EE. Results from a study reported by Ohsfeldt et al. indicated that the 1-year cost of HT for treating VMS was approximately $300 greater than the cost associated with no treatment [47]. It is not clear how results from this Canadian analysis would apply to the United States since health care is much less expensive in that country.

**Table 2:** Baseline Costs for a Pharmacoeconomic Model of Vasomotor Symptoms

**Drug acquisition costs**	
Norethindrone acetate/ethinyl estradiol	$357
Conjugated estrogen/medroxyprogesterone	$474
**Therapy initiation**	
Two physician visits	$132
**Breakthrough bleeding at 3 months (or continued spotting at 6 months)**	
Endometrial biopsy	$198
Pathology and laboratory fees	$147
**Telephone call to physician**	
Spotting at 3 months	$16
**Moderate to severe vasomotor symptoms**	
Two physician visits	$132
90-day supply of clonidine	$31

Adapted with permission from Botteman et al. [45].

As noted, the published results from the WHI have dramatically reduced the use of estrogen and HT by menopausal women. Results from the WHI prompted reanalysis of the cost-effectiveness of HT by investigators at the Stockholm School of Economics. Generalization of results from this analysis may be limited because Sweden has a national socialized medical system. However, the authors suggest that HT remains a cost-effective therapeutic strategy for women with menopausal VMS compared with no therapy [47].

### SSRIs and SNRIs

Selective serotonin reuptake inhibitors (SSRIs) and serotonin-norepinephrine reuptake inhibitors (SNRIs) have received increased attention for the management of VMS in nondepressed menopausal women [48]. As yet, there have been no pharmacoeconomic analyses of any agents in these classes for this indication. Results from a recent systematic review of published economic evaluations of interventions for depression indicated that SSRIs and SNRIs are more cost-effective than older antidepressant medications (eg, tricyclic antidepressants) owing to their greater efficacy and decreased side-effect profile [49].

### Other prescription medications

Other prescription medications approved for use in conditions not associated with menopause-related VMS have demonstrated varying degrees of efficacy [48]. The economic advantage for some of these medications is that they have been on the market for a number of years. Given the understanding that the VMS are the result of a dysfunction in thermoregulatory circuitry, new nonhormonal therapies that selectively target the serotonin and norepinephrine pathways, without the involvement of other pathways, seem likely to become the next generation of care for the management of VMS.

### CAM treatments

Many symptomatic menopausal women are likely to treat themselves before consulting a medical practitioner, thinking that "natural" products are safer and the ingredients more pure than synthetic agents. Cost analysis was carried out for CAM treatments that women would commonly find through a basic search on the Internet using the terms *complementary medicine *and *hot flash *as search parameters. The most common CAM treatments that emerged were products containing individual and compounded formulas of herbs, isoflavones, and dietary supplements that promised to alleviate menopause-related hot flushes and night sweats, irritability, sleeplessness, mood swings, weight gain, headaches, insomnia, depression, menstrual irregularities, fatigue, and loss of sexual desire. These formulations also claim to promote mental clarity, increase energy levels, and improve physical performance [50-53]. The initial cost of a single product ranged from $19.95 to $58.00 per month (Table 3). A key limitation in the analysis of these products is that their clinical efficacy has generally not been documented by results from controlled clinical trials. It has also been noted that any benefits associated with herbal supplements may occur more slowly than those achieved with traditional medications [54]. Comparison of 1-year costs of CAM treatments versus HT indicated that three of seven alternative treatments were more expensive than traditional therapy (Table 3).

**Table 3:** Complementary and Alternative Medicine Costs Over 6 Months

**Product**	**Main Ingredients**	**Cost ($US) at 1 Month**	**Cost ($US) at 3 Months**	**Cost ($US) at 6 Months**
Femforte^®^	Black cohosh, soy isoflavones, androstenedione, chaste berry	58.00	174.00	348.00
MACA750™	Organic maca root	19.95	59.85	119.70
Promensil™	Red clover	24.95	64.90	129.80
Remifemin^®^	Black cohosh extract equivalent to 20 mg dried *Cimicifuga *rhizome	49.99	78.98	157.96
Sleep & Slim™	L-glutamine, L-lysine HCl, magnesium citrate, L-ornithine, glysine, L-arginine, collagen, vitamin B_6 _(pyroxidine HCl), L-carnitine, vitamin B_3 _(niacin), aloe vera, ascorbic acid, citric acid, sodium benzoate, potassium sorbate, carrageenan	49.95	149.85	299.70
Effisoy™	AglyMax: fermented soy germ extract	29.95	79.35	149.75
Hot Flash, Non-GMO Soy	Geneistein-rich soy concentrate, black cohosh root extract, dong quai root extract, licorice root extract, vitex berry extract	45.98	137.94	275.88

Note: Three-and 6-month costs that are lower than 3 and 6 times the 1-month costs for certain items reflect bulk prices for the items.

## Conclusion

Menopause-related VMS are very common and can be associated with a high patient and societal burden. These symptoms result in high direct and indirect costs and significantly reduced QOL. Current treatments for VMS include HT, prescription medications developed for other indications, and CAM treatments. Short-term HT has been shown to be cost-effective for the management of VMS, but the publicity given the WHI has substantially decreased the use of these treatments. The physiology underlying VMS is complex and not fully understood, but it is clear that alterations in noradrenergic and serotonergic mechanisms during hypothalamic thermoregulation are involved in their development. A significant unmet need remains for menopause-related VMS treatment options. Among women who are eligible for the treatment of menopause-related VMS, 80% do not seek treatment, receive inadequate counseling, or do not have access to local medical aid [39]. The development of therapies that specifically target VMS may provide high efficacy and reduced risk of serious and potentially costly adverse events, thus increasing the overall cost-effectiveness of therapy.

## List of abbreviations

BMI Body mass index

CAM Complementary alternative medication

CEE/MPA Conjugated estrogens plus medroxyprogesterone acetate

HRQOL Health-related quality of life

HT Hormonal therapy

NA/EE Norethindrone acetate plus ethinyl estradiol

QALY Quality-adjusted life-years

QOL Quality of life

SF-36 36-Item Short Form Health Survey

SNRI Serotonin-norepinephrine reuptake inhibitor

SSRI Selective serotonin reuptake inhibitor

SWAN Study of Women's Health Across the Nation

UQOL Utian Quality of Life

VMS Vasomotor symptoms

WHI Women's Health Initiative

WHQ Women's Health Questionnaire
